# Dual Implications of Nanosilver-Induced Autophagy: Nanotoxicity and Anti-Cancer Effects

**DOI:** 10.3390/ijms242015386

**Published:** 2023-10-20

**Authors:** Lidia Strużyńska

**Affiliations:** Laboratory of Pathoneurochemistry, Department of Neurochemistry, Mossakowski Medical Research Institute, Polish Academy of Sciences, 5 Pawińskiego str., 02-106 Warsaw, Poland; lidkas@imdik.pan.pl

**Keywords:** autophagy defect, AgNPs, cancer, cytotoxicity, lysosomes, ER stress, cell death, apoptosis

## Abstract

In recent years, efforts have been made to identify new anti-cancer therapies. Various types of nanomaterials, including silver nanoparticles (AgNPs), are being considered as an option. In addition to its well-known antibacterial activity, AgNPs exhibit cytotoxic potential in both physiological and cancer cells by inducing stress-mediated autophagy and apoptotic cell death. A rapidly growing collection of data suggests that the proper regulation of autophagic machinery may provide an efficient tool for suppressing the development of cancer. In this light, AgNPs have emerged as a potential anti-cancer agent to support therapy of the disease. This review summarizes current data indicating the dual role of AgNP-induced autophagy and highlights factors that may influence its protective vs. its toxic potential. It also stresses that our understanding of the cellular and molecular mechanisms of autophagy machinery in cancer cells, as well as AgNP-triggered autophagy in both normal and diseased cells, remains insufficient.

## 1. Introduction

The general toxicity of nanomaterials (NMs), including AgNP-induced toxicity, has been previously described, and underlying molecular mechanisms, such as oxidative stress, apoptotic/necrotic cell death, and autophagy, have been discussed [1,2,3]. 

Autophagy has been characterized as a self-degrading catabolic process that utilizes cytoplasmic macromolecules and damaged organelles to recover energetic substrates in cells in order to adapt to stressful conditions [4]. The important role of this mechanism in the toxicity of nanomaterials has been emphasized [1,3,5]. Autophagic structures and autophagy-promoting proteins have been detected across a range of cell culture models exposed to AgNPs [6,7,8,9,10,11,12,13,14,15]. Ultrastructural and/or molecular markers of autophagy have also been reported in animal models, e.g., in the liver of rats after an intraperitoneal administration of AgNPs [16], and in the brains of adult rats [17] and immature rats [18] which were orally exposed to AgNPs. However, the cellular and molecular mechanisms of AgNP-induced autophagy and its role in different cells under exposure to AgNPs are not yet well established. 

In addition to being described in terms of nanotoxicity, autophagy has been linked to various pathological conditions, including cancer [19,20]. Dysregulation of this process is known to affect many mechanisms during tumor formation, development, and metastases, and has been reported to promote both cancer cell survival and death, as well as to control other cancer-related processes, such as oxidative stress, inflammation, and immune response [21,22]. Although the contribution of autophagy to oncological diseases is unquestionable, our understanding of the underlying mechanisms is still far from complete. 

In recent decades, considerable efforts have been made to search for new anti-cancer therapies. Various types of nanomaterials, including AgNPs, are being considered as an option [23]. The therapeutic potential of AgNPs is based on their ability to induce mammalian cell death in both healthy and cancerous tissues, depending on the biological characteristics of the targeted cell. Therefore, they can potentially be used as anti-cancer agents to support standard therapies by inducing oxidative stress-mediated autophagy and/or apoptosis. Current views on the possibility of using AgNPs in oncotherapy have recently been discussed in context of developing novel effective therapeutic tools. The ways to target tumor tissue, interactions between AgNPs and tumor cells, and intracellular molecular effects have been summarized in an effort to characterize realistic opportunities for using AgNPs in cancer treatment [24]. 

The anti-cancer therapeutic potential of AgNPs may be based on the induction of cell death via several mechanisms. After their uptake by endocytosis-related mechanisms, AgNPs accumulate in lysosomes, the acidic environment of which facilitates the release of silver ions from their surfaces. Both forms, particulate and ionic, interact with mitochondria, leading to the formation of reactive oxygen species (ROS) and consequently oxidative stress (OS), which activates cellular pathways leading to cell death. ROS interact with genomic DNA in tumor cells to induce DNA damage and activate the tumor suppressor P53 and the P53-dependent apoptotic pathway. Mitochondria targeted by AgNPs lose their membrane potential and become damaged. Cytochrome c, released from damaged mitochondria into the cytoplasm, triggers the classic apoptotic pathway. Moreover, AgNP-induced ROS are capable of inducing endoplasmic reticulum (ER) stress and sustained autophagy, both of which may result in apoptosis and/or autophagic cell death (reviewed in [24]). 

To advance the current knowledge of the therapeutic potential of AgNPs, this review highlights autophagy as one of the biochemical mechanisms of nanotoxicity and describes the role of AgNP-induced autophagy in cell survival or death. It also provides insights into previous research on AgNP-induced autophagy to establish the determinants of its toxic/protective profile in the cell, which may be useful for the development of AgNP-based strategies in cancer treatment. This review also concludes that many critical issues relating to the use of AgNPs in such an onco-therapeutic approach require clarification.

A search of databases, such as PubMed and Scopus, was conducted to extract relevant data. Keywords for database searches included the following: metal nanoparticles and autophagy, AgNPs toxicity, AgNPs and autophagy, cancer and autophagy, and AgNPs and cancer therapy. Experimental and review articles on AgNP-induced autophagy in various cell types and in vivo models were included. Studies on nanoparticles other than AgNPs were excluded. 

## 2. The Autophagy Process and Its Importance for the Cell

The proper function of cells significantly depends on the maintenance and renewal of the entire proteome. The ubiquitin-proteasomal system and the autophagy-lysosomal system are cellular degradation systems which act to ensure proper cellular operation. In eukaryotic organisms, a lysosomal pathway is represented by autophagy which is triggered by the cell under stressful conditions. By the degradation of misfolded proteins, damaged organelles, and cytosolic components in autophagolysosomes, this multi-step complex mechanism supplements energy for essential cellular processes in order to increase metabolic activity and maintain cellular homeostasis [4]. 

**Figure 1:**
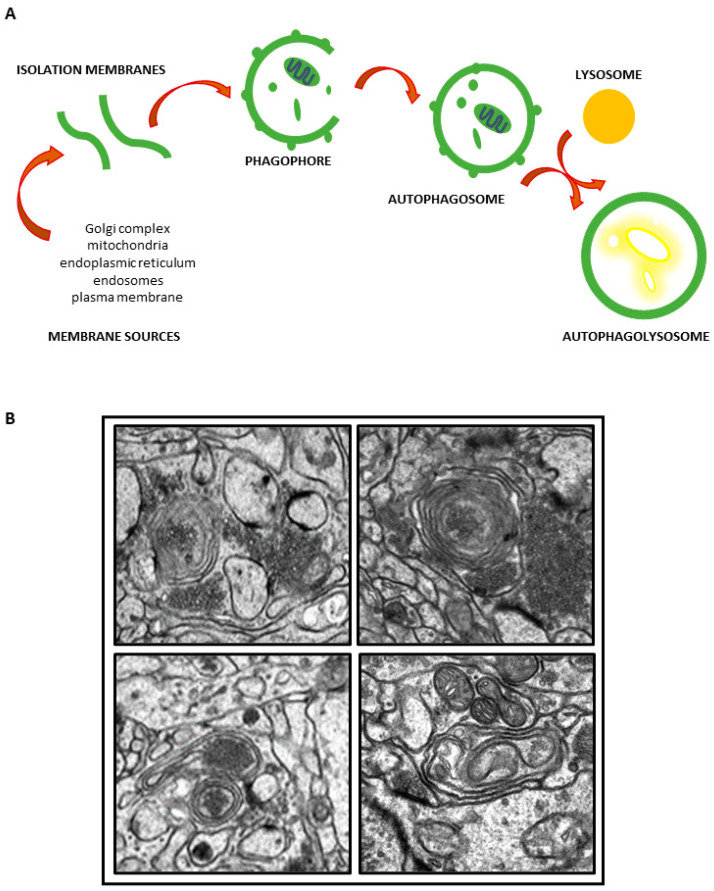
Schematic representation of autophagy flux (**A**); autophagic structures in brain of AgNP-exposed rats (**B**). TEM images from author’s own experimental resources.

The ultrastructural features indicating the induction of autophagy in the cell are isolation membranes created in the cytoplasm. These double-membraned structures are referred to as phagophores and are generated from various cellular sources, such as the endoplasmic reticulum (ER), plasma membrane, or outer mitochondrial membrane (for reviews, see [19,25]). These membranous structures elongate and sequester cytosolic contents, such as damaged proteins or organelles, and form spherical autophagosomes which then fuse with lysosomes to form autophagolysosomes (Figure 1A,B). Lysosomal enzymes degrade the encapsulated material, generating energy and new components necessary for cell homeostasis [26]. 

Autophagy, also referred to as macroautophagy, is generally considered to be a protective mechanism which is activated to allow the cell to survive under various stressful stimuli, including nutrient or energy deprivation, endoplasmic reticulum (ER) stress, hypoxia, immune signals, redox stress, and mitochondrial damage, each of which may stimulate autophagy through diverse signaling pathways which have been reviewed in detail [19,25]. To provide an overview of the major molecular components of the core autophagic machinery shared by different stimuli, several groups of key molecules involved in the subsequent stages of this process, such as initiation, elongation, maturation, and fusion with the lysosomes, should be identified. During the early steps of autophagosome formation, two main macromolecular complexes are implicated. These complexes are the beclin 1/phosphatidylinositol 3-kinase (PI3K complex) and the unc-51-like kinase 1/2 (ULK1/2) complex. The elongation process requires the Atg9 protein, as well as two ubiquitin-like reactions of the Atg12-Atg5 complex, and the microtubule-associated protein 1 light chain 3 (MAP1-LC3 protein), that mediate the formation of autophagic structures. The maturation of autophagosomes and fusion with lysosomes involves proteins such as ESCRT, SNAREs, Rab7, and the class CVps proteins [19,21,25,27]. In addition to macroautophagy, selective autophagy is defined as any process where different organelles, such as ER, mitochondria, and ribosomes, are cleared by autophagy. Examples include ERphagy, mitophagy, and ribophagy, respectively (for a review, see [28]).

Mechanistically related to ER stress, autophagy is potently activated downstream of the cell’s stress defense mechanism, which is known as the unfolded protein response (UPR) to counteract ER stress [29]. This cellular mechanism of adaptive response is initiated by three regulatory proteins: inositol requiring 1 (IRE1), PKR-like ER kinase (PERK), and activating transcription factor 6 (ATF6), which under normal conditions are rendered inactive by the chaperone protein GRP78 (glucose-regulated protein 78 kDa, also known as BiP). 

The UPR-mediated adaptive response increases the autophagy process and associated protein degradation in order to promote the clearance of damaged proteins [30]. The upregulation of genes encoding major UPR regulators upon ER stress conditions also leads to the transcriptional overexpression of genes related to cell survival or death. All three UPR branches, IRE1, PERK, and ATF6, can directly induce and regulate autophagy and autophagosome formation during ER stress via the activation of multiple genes that regulate the expression of several autophagy inhibitors [31]. 

Under stress conditions, the cell initially triggers defense mechanisms that help to adapt to the stress. The cell fate is then determined according to the stress conditions. Autophagy is generally considered as a pro-survival mechanism. When upregulated, autophagy increases catabolism and the recycling of damaged components, thereby sustaining cell homeostasis. When the process of autophagy activation dangerously increases beyond a critical threshold, the cell can be directed to the apoptotic pathway. Stress-adaptive autophagy or, alternatively, apoptosis, can be triggered by common upstream signals [32]. Alternatively, due to the accumulation of autophagy-related substances, autophagic cell death (ACD) may occur with features characterized by excessive sequestration of autophagosomes in the cytoplasm, which takes on a vacuolated appearance. The presence of a large number of autophagic structures in dying cells is helpful in distinguishing between autophagic and apoptotic cell death [33]. 

Currently, the dominant view is that basal autophagy is a physiologically important mechanism, induced by the cell for beneficial effects. The upregulation or dysfunction of this process is closely related to aging and various disease conditions. Even then, the autophagy may be protective to some extent [27]. 

### The Role of Autophagy in Cancerous Process

As outlined above, the inability of the cell to properly regulate autophagy is associated with aging and is also implicated in the pathogenesis of various disorders, such as neurodegenerative diseases, type 2 diabetes, Crohn’s disease, and cancer [19,20,27]. Therefore, the proper regulation of the autophagy process has been suggested as a novel therapeutic strategy for the treatment of these diseases [34,35], particularly cancers [20,22], where clinical studies attempting to inhibit autophagy are being conducted. 

In a normal cell, autophagy is an important protective mechanism that prevents the transition into a cancer-initiating cell by maintaining cell homeostasis through the removal of oncogenic protein substrates, toxic unfolded proteins, and damaged organelles [36]. The dysregulation of this machinery favors oncogenic processes where autophagy plays various roles, which often compete with each other, depending on many factors. 

Studies indicate that increased autophagy in cancer cells helps them to overcome stressful conditions, such as hypoxia and nutrient deprivation inside the tumor, facilitating their survival and growth [37]. Among other processes, autophagy regulates mitochondrial functions by supplying energetic substrates such as amino acids and fatty acids. Selective autophagy, mitophagy, protects cells from oxidative stress by the elimination of ROS-generating damaged mitochondria [38] The manipulation of autophagic flux is therefore expected to prevent cancer development. Blocking the autophagy/mitophagy process results in an increased rate of production of ROS and mitochondrial damage, which is followed by the devastating effect of oxidative stress on protein and DNA integrity, leading to the suppression of tumor growth [39]. For these reasons, inhibitors of the late stages of the autophagy process such as chloroquine or hydroxychloroquine are currently used in clinical trials to treat various types of cancer (for a review, see [40]). 

However, increasing evidence suggests that the role of autophagy in cancer is more complex [38]. In an early phase of the disease, autophagy has been linked to both autophagy-related cell death and tumor promotion and growth (for reviews see: [20,41]). Autophagy may also be protective in the progressive phase of the disease by increasing the survival of cancer cells during detachment from tumor, thus facilitating metastasis [42]. 

Autophagy can increase tumor growth and survival in different ways, including support of the metabolic adaptation of cancer cells and assisting the escape of immune system effects via the degradation of major histocompatibility complex class I (MHC-I) [43]. Tumor suppression during autophagy activity is largely due to the removal of oncogenic protein substrates, toxic unfolded proteins, and damaged organelles [20]. In advanced cancers, opposing roles of autophagy have been also described regarding metastasis processes. Beneficial effects rely on the support of the resistance of cancer cells to nutrient deprivation and resistance to detachment-induced cell death. Metastasis suppressive effects include the autophagic degradation of the glycolysis mediator (PFKFB3) and maintenance of genomic stability [43]. Autophagy may also play an important role in the maintenance of cancer stem cells, which are a small subpopulation of cells with the ability to self-renew and differentiate, thus contributing to tumor metastasis [44].

The role of autophagy in cancer development and progression still raises a number of questions [20]. It appears that the function of this mechanism in cancer biology is context-dependent and highly influenced by the cancer subtype, tumor microenvironment, disease stage, and exposure to external stimuli [22,38]. The complex molecular mechanisms of autophagy in cancer cells and its diverse roles in different phases of the disease require further investigation in order to consider the application of NP-based therapies. 

## 3. Nanosilver-Induced Autophagy—Toxic or Protective Role?

### 3.1. Ultrastructural and Molecular Characteristics Indicative of Autophagy Induction by AgNPs In Vitro and In Vivo

The activation of autophagy processes has been previously reported to be a common mechanism of toxicity induced by various nanomaterials including AgNPs [3,5,45]. The activation of autophagy by AgNPs has been observed in vitro in various cells and cell lines of physiological origin (Table 1), such as monocytic THP-1 cells [46,47], mouse embryonic fibroblasts [4,48], mouse hippocampal neuronal cell line (HT22 cells) [10], HEK293T human embryonic kidney cells [8], and HC11 mammary epithelial cells [49], as well as in cancer-derived cell lines (Table 2), including the human liver cancer cell line HepG2 [6], HeLa cells [50], the human neuroblastoma cell line SH-SY5Y [7], or A498 renal carcinoma cells [8]. The induction of autophagy has also been confirmed in animal models of AgNP exposure [7,16,17,18] (Table 1) (Figure 1B). 

The autophagy induced by AgNPs in vitro is typically characterized by enhanced autophagosome formation and the presence of autophagic vacuoles containing partially degraded cytoplasmic material as identified by transmission electron microscopy (TEM). For instance, in the AgNP-treated HT22 cell line which is derived from primary-cultured murine hippocampal neurons, typical autophagic vacuoles were found that included double-membrane phagophores, autophagosomes, and autolysosomes with cytoplasmic materials inside [10]. Similarly, autophagosome formation was identified in the NIH 3T3 mouse embryonic fibroblast cell line [48] and the PANC-1 pancreatic adenocarcinoma cell line [11]. 

In fluorescence microscopy studies, numerous acidic vesicles known as acidic vesicular organelles (AVOs) were observed in the perinuclear region of the cytoplasm of AgNP-treated cells in a significantly higher percentage compared to untreated cells [48]. Monodansylcadaverine (MDC), a specific autolysosome marker, was used to demonstrate its increased incorporation into human osteosarcoma (OS) cells after autophagy stimulation by biologically synthesized AgNPs. This indicates an increase in the number of autophagic vesicles [51]. Typically, the activation of autophagy in various cells has also been demonstrated by the overexpression of molecular markers such as LC3-II [6,7,8,10,48,51]. The expression of other autophagy-related proteins such as beclin-1 has been reported to be stable [10,48] or increased [4,12,13,14,49,51,53], and the activity of the lysosomal peptidase, cathepsin B, has been noted to be decreased [9].

Similar to the results of in vitro studies, an ultrastructural analysis of liver tissue obtained from rats exposed to a single dose of AgNPs (500 mg/mg b.w.) revealed double-membrane autophagosomes with engulfed damaged organelles and autolysosomes containing significant amounts of cellular debris [16]. Membranous structures that are characteristic of the autophagy process were found to be present in the brain tissue of immature rats [17,18] and adult rats [50] exposed to a low dose of AgNPs (0.2 mg/kg b.w.) (Figure 1B). Some of these structures are indicative of the initial steps of the formation of autophagosomes, while others contained cellular remnants, indicating the presence of mature forms of autophagosomes. Diverse ultrastructural forms of mitochondria were also found to accompany typical autophagic structures, such as disturbed and fragmented mitochondria [17] and elongated mitochondria [18], the latter of which are a known characteristic of ER stress-induced autophagy and represent a protective process of mitochondrial elongation triggered by the cell via the mechanism of stress-induced mitochondrial hyperfusion (SIMH) [54]. 

There is a close relationship between autophagy and mitochondria, which undergo morphological and molecular remodeling of their shape, form, and biocomposition in response to stress. Mitochondrial fragmentation and the depolarization of mitochondrial membranes are major factors that trigger the process of autophagy, which is specifically referred to as mitophagy [55]. Interestingly, prolonged exposure to AgNPs has previously been shown to disrupt mitochondrial function in the brain of AgNP-exposed adult rats as expressed by a decrease in the mitochondrial membrane potential ΔΨm [17], which is an index of mitochondrial condition. The increased number of lysosomes observed in the brain of AgNP-treated rats may also be indicative of the induction of autophagy, reflecting an increased capacity of the cell to degrade and recycle damaged biomolecules [18]. 

In addition to specific morphological characteristics, the upregulation of molecular markers has also confirmed the presence of AgNP-induced autophagy in vivo. The overexpression of proteins such as beclin 1 and LC3-II, which are involved in the formation of autophagosomes in the early stages of autophagy, was found in the brains of AgNP-exposed adult rats [17]. Also, the expression of LC3-II was found to increase significantly in the liver tissue of AgNP-exposed rats shortly after exposure [16]). Being an integral part of autophagosomal membranes, these proteins are widely used to quantify autophagy activity [56]. 

### 3.2. Interference of AgNPs with Autophagic Flux in Different Models of Exposure

An important feature of autophagy induced by AgNPs is defective flux associated with the blockade of the last step of this process, which is the fusion of the autophagosome with the lysosome and the subsequent degradation of cellular debris. This mechanism is not unique to AgNPs but has also been demonstrated for other types of nanoparticles, such as graphene oxide quantum dots [57] and silica NPs [58].

Existing data indicate that AgNPs are taken up into various cells and accumulate in the cellular lysosomal compartment. Lysosomal vesicles containing nanoparticle aggregates have been shown to exist in vitro in various cells such as non-cancerous HC11 cells [49], cancer-derived HeLa cells [50], and human liver-derived hepatoma HepG2 cells, where they were observed in cytosolic vesicles 24 h post-exposure [6]. In addition, in animal models of exposure, AgNPs were found to accumulate in lysosomes of rat liver [16] and in the lysosomal compartment of brain endothelial cells and neurons [17]. 

The overloading of lysosomes with endocytosed AgNPs can result in the permeabilization of the lysosomal membrane, internal alkalinization, and inactivation of lysosomal enzymes, ultimately leading to lysosomal dysfunction. It has been suggested that lysosomal impairment is the primary cause of the AgNP-induced blockade of autophagic flux [1]. Moreover, the permeabilization of lysosomal membranes by cathepsins can lead to apoptotic cell death triggered by the permeabilization of the outer mitochondrial membrane [59]. Nanomaterial-induced dysfunctional lysosomes have been linked to the execution of cell death in the form of apoptosis, pyroptosis, ferroptosis, or necroptosis. The dysregulation of lysosomal function by nanomaterials is currently considered to be a main cause of their nanotoxicity (for a review see: [60]).

Regarding AgNPs, their presence in the lysosomal compartment leads to the disruption of lysosomal functions followed by the blockade of induced autophagy [9,46] and destabilization of the autophagy-lysosomal system [6]. Furthermore, the lysosomal hydrolases known as cathepsins have been reported to correlate with the formation of inflammasomes and release of IL-1β from human blood monocytes [61].

Morphologically, the disruption of lysosomal trafficking and fusion results in the accumulation of autophagic and lysosomal vacuoles inside the cell. In turn, one of the molecular markers of autophagic flux is p62 protein, also referred to as sequestosome 1 (SQSTM1), which is a ubiquitin-binding scaffold protein that links ubiquitinated proteins to the autophagic machinery to enable their degradation in the lysosomes. Since this protein is degraded by autophagy, its level decreases after the induction of autophagy, while it accumulates inside the cell during the inhibition of autophagy [62]. An increase in the LC3-II/LC3-I ratio and accumulation of p62 are considered indicative of a defective autophagic flux [1].

AgNPs have been shown to block the degradation of the autophagy substrate p62 and to induce the accumulation of autophagosomes in monocytic THP-1 cells, as well as to produce lysosomal alkalization and the destabilization of the lysosomal membrane. The blockade of AgNP-induced autophagy and lysosome dysfunction thus leads to the inhibition of the differentiation of monocytes into macrophages [46]. Increased levels of p62 have also been reported in AgNP-treated HEK293T human embryonic kidney cells and A498 renal carcinoma cells [8], as well as in A549 human lung adenocarcinoma cells, where the blockade of autophagy occurs due to lysosomal dysfunction [52]. According to Mishra et al. [6], AgNPs can cause cell injury by destabilizing the autophagy-lysosomal system in HepG2 cells, thus leading to NLRP3 inflammasome-dependent caspase-1 activation, ER stress, release of lactate dehydrogenase, and apoptosis. 

In animal models of exposure, some markers of autophagy blockade have been reported. The expression of Rab7, a protein involved in the process of autophagosome maturation and autophagolysosome formation [63], was not elevated in the brain tissue of AgNP-treated rats, although other molecular markers of autophagy were found to be increased, indicating the activation of this process [17]). Since Rab7 is involved in the maturation of autophagosomes, its deficiency may suggest an inefficient autophagic process. 

It is worth noting that a defective autophagic flux deprived of the last step of lysosomal degradation of cellular remnants has been linked to aging and certain disorders [27,64]. Therefore, the interference of AgNPs with physiological autophagic flux definitely increases their cytotoxicity [1]. This is a desirable characteristic of nanotools designed to treat cancer. However, the data summarized in Table 1 and Table 2 clearly show that the molecular markers of autophagy blockade are not commonly observed in the listed physiological and cancerous cell types exposed to AgNPs. Importantly, the blockade of autophagy does not occur in several cancer cell lines, such as HeLa cells [50], SH-SY-5Y [7], human OS cells [12], and breast cancer cells [14], thus promoting cell survival. Do these results indicate that AgNPs do not always interfere with autophagic flux, or do we need profound studies on the factors upon which it depends? Unfortunately, considering the currently available data, it is difficult to draw conclusions about any dependencies.

### 3.3. Evidence for Pro-Death and Pro-Survival Profiles of AgNP-Induced Autophagy in Normal Cells In Vitro and In Vivo

Dysfunctional autophagy, defined as massive autophagy induction, dysregulation, or the blockade of autophagic flux, is recognized as a potential mechanism of cell death by apoptosis or ACD. Although autophagy often accompanies cell death and there is crosstalk between autophagy and other cell death pathways, the mechanisms underlying autophagy-induced cell death are not clearly defined [33].

Certainly, under the stressful conditions of AgNP exposure, autophagy is primarily induced by the cell as a defense mechanism. However, the reports regarding AgNP-induced autophagic blockade raise a question about the effectiveness of this primarily protective mechanism, as well as to what extent the cell death observed in exposed cells is the primary effect of AgNPs acting via other molecular mechanisms. There is also the question of the extent to which cell death is a result of an ineffective autophagy process. 

A series of in vitro and in vivo studies have been conducted over the last few years on the molecular mechanisms of AgNP-induced nanotoxicity. It has been reported that exposure to AgNPs results in the disruption of mitochondria [17,65], and the overproduction of ROS with subsequent oxidative stress [2,66], as well as ER stress [1,18,47,67,68]. An excess of ROS, not counterbalanced by cellular defense mechanisms, can ultimately lead to cell death by damaging organelles and biomolecules, such as DNA, proteins, and lipids. Both the overproduction of ROS and oxidative stress are known to induce apoptosis and autophagy. This critical relationship between mitochondrial dysfunction, oxidative stress, and autophagy in the brains of rats exposed to AgNPs has been previously characterized [17]. 

Autophagy triggered by exposure to AgNPs may reflect a stress-adaptive response induced by the cell against nanoparticle-induced harmful effects, such as the efficient removal of oxidatively damaged proteins and/or organelles, or, alternatively, may be deleterious itself, increasing cellular dysfunction and leading to the cell death by crosstalk with the apoptotic machinery. Experimental studies have provided evidence supporting both of these mechanisms.

Results of in vitro studies indicate that in parallel with autophagy, AgNPs can further trigger apoptosis in the hippocampal neuronal cell line HT22 by upregulating caspase-3 and the pro-apoptotic Bax protein simultaneously with the downregulation of the anti-apoptotic Bcl-2 protein. Moreover, AgNP-induced cytotoxicity was found to be mediated by both autophagy and apoptosis via the PI3K/AKT/mTOR signaling pathway [10]. Also, in the mouse embryonic fibroblast treated with AgNPs, an increased percentage of apoptotic cell death was found to occur, as measured by flow cytometry with the Annexin V apoptosis detection kit. Significant nuclear fragmentation was also observed, as well as the activation of PARP and caspase 3 [48]. 

In vivo studies of the brain tissue of adult AgNP-exposed rats have shown that autophagy is linked to synaptic degeneration as indicated by ultrastructural and molecular markers [69]. The ultrastructural characteristics, in the form of disrupted synaptic membranes and free synaptic vesicles located extrasynaptically in the neuropil, have been found to be specific for AgNPs but not for ionic Ag. Concentrically layered autophagy structures were found to contain fragmented organelles, such as clusters of synaptic vesicles and mitochondrial remnants. Simultaneously, the expression of molecular markers of synaptic density, such as synapsin I, synaptophysin, and PSD-95, were found to be decreased, thus confirming the loss of synapses [69]. 

In turn, autophagy of a protective nature has been shown to counteract AgNP-induced cell death in a mouse mammary epithelial cell line (HC11) by acting via an Akt/AMPK/mTOR pathway associated with cellular oxidative stress and mitochondrial injury [49]. Similarly, in vivo studies by [16] indicate that autophagy is the main protective mechanism activated to counteract the hepatotoxicity of AgNPs. However, according to the authors, the inability of the cell to properly maintain the autophagic mechanism once activated, is associated with energy deficiency, and may lead to the apoptotic death of hepatocytes and subsequent liver dysfunction. This conclusion was drawn based on the time-course of AgNP-induced autophagy, the increased expression of apoptosis markers such as caspase-3 and TUNEL-positive cells, and energy failure in the liver of rats exposed to a single dose of nanoparticles (500 mg/kg b.w.). 

A pro-survival effect of ER stress-related autophagy has been also reported in the brain tissue of rats subjected to a low dose of AgNPs [17]). This study did not exhibit the morphological or molecular characteristics indicative of apoptosis induction. A low Bax/Bcl-2 ratio (1.15) was observed. The toxic effects of AgNPs were probably counteracted by the enhanced biodynamics of mitochondria as evidenced by the elongated structures of these organelles. The maintenance of physiological levels of ATP and the lack of molecular and ultrastructural markers of apoptosis confirmed the effective compensation. Similarly, dynamic mitochondrial biogenesis resulting in mitochondrial elongation via the SIMH mechanism, has been observed in the brain tissue of immature rats exposed to a low dose of AgNPs [18]. According to the authors, protective mitochondrial elongation is a cellular defense reaction against AgNP-induced ER stress that is activated to suppress pathological mitochondrial fragmentation and to promote mitochondrial functionality [70]. In the study of Dąbrowska-Bouta et al. [18], the remodeling of mitochondrial quality was considered to facilitate recovery from AgNP-induced ER stress by increasing cellular energetic capacity via the PERK arm of the UPR mechanism.

The overexpression of the C/EBP Homologous Protein (CHOP), another molecule downstream UPR pathway, was also identified in AgNP-exposed animals, indicating the pro-apoptotic output of ER-stress-induced UPR. However, a microscopic analysis did not confirm the neuronal death via the apoptotic pathway [18]. As reviewed by Mao et al. [1], ER stress may play an important role in modulating the interplay between AgNP-induced autophagy and apoptosis.

The analysis of Table 1 shows that autophagy is accompanied by apoptosis or other types of cell death in in vitro models of AgNP exposure, while in animal models it is often linked to the protective defense mechanisms of the organism. 

### 3.4. Evidence for Pro-Death and Pro-Survival Profiles of AgNP-Induced Autophagy in Cancer Cells In Vitro and In Vivo

The vast majority of in vitro studies using cancer-derived cell lines indicate that AgNP-induced autophagy leads to cytotoxic effects. Cytotoxicity was observed among other effects in PANC-1 adenocarcinoma pancreatic cancer cells as a result of stimulated apoptotic and autophagic cell death. In PANC-1 cells, AgNPs significantly induce increased levels of the tumor suppressor p53 protein, an increased ratio of pro-apoptotic Bax/anti-apoptotic Bcl-2 proteins, as well as increased levels of necroptosis- and autophagy-related proteins: RIP-1, RIP-3, MLKL, and LC3-II [11]. Damage to A549 human lung adenocarcinoma cells has been reported to be caused by AgNP-induced lysosomal dysfunction, which plays a principal role in the defect of autophagic flux [52]. Such lysosomal dysfunction is the result of the AgNP-induced reduction of transcription factor EB (TFEB) expression, which is a major regulator of lysosomal biogenesis and a regulatory factor of autophagic genes. The cytotoxic effects of AgNPs towards cancer cells was found to be potentiated by designing nanocomposites of AgNPs with cisplatin and reduced graphene oxide (rGO–Ag-NPs). This nanotool was found to significantly increase the accumulation of autophagosomes and autophagolysosomes in HeLa cells, which are associated with the generation of ROS and cell death [71]. Furthermore, the synergistic cytotoxic effect of salinomycin and Ag-NPs was shown in A2780 ovarian cancer cells, where the induction of massive autophagy causes the accumulation of autophagolysosomes, which further leads to mitochondrial dysfunction and cell death [72]. AgNPs capped with an exopolysaccharide (EPS) have also been shown to exert a cytotoxic effect against SKBR3 breast cancer cells. A proteomic analysis highlighted important pathways involved in AgNP-EPS toxicity, including ER stress, oxidative stress, and mitochondrial impairment, each of which triggers cell death through apoptosis and/or autophagy activation [14]. 

Interestingly, in parallel with the initiation of cell-death, AgNP-induced autophagy can also protect cancer cells from nanotoxicity as described for normal cells (Section 3.3). Supporting evidence comes from the study of Lin et al. [50], where PVP-coated AgNPs were found to induce protective autophagy, thereby increasing the survival of both B16 melanoma cells and HeLa cells. The normal degradation of lysosomal cargo occurred and the ability of AgNPs to block normal autophagic flux was not observed in this study. Only the inhibition of autophagy by the chemical inhibitor of autophagy, wortmannin, significantly stimulated apoptotic cell death. The use of wortmannin in a B16 melanoma mouse model also significantly enhanced the antitumor efficacy of AgNPs by increasing apoptosis and stimulating tumor shrinkage [50].

Similarly, autophagy induced in human OS cells and huh7 hepatocellular carcinoma has been shown to function as a pro-survival strategy in cancer cells [51]. The biologically synthesized AgNPs (bAgNPs) were found to induce ROS-dependent apoptosis concomitantly with the upregulation of pro-survival autophagy via the c-Jun N -Terminal kinase (JNK) signaling pathway. This mechanism tends to inhibit apoptosis by limiting enhanced ROS production [51]. Li et al. [7] also suggests the existence of a cytoprotective profile of AgNP-induced autophagy in SH-SY-5Y cells. Only the inhibition of autophagy by the addition of chloroquine or silencing of beclin-1 significantly enhances the cytotoxicity of AgNPs, suggesting a protective role of autophagy mediated via the Ca^2+^/CaMKKβ/AMPK/mTOR pathway. The results of the study by Jeong et al. [15] suggest that hypoxic conditions in A549 cancer cells reduce the cytotoxic potential of AgNPs by attenuating AgNP-induced apoptosis via the HIF-1α-mediated autophagy pathway. 

The key mediator of Ag NPs-induced cytoprotective autophagy may be TFEB. Autophagy induced by AgNPs in HeLa cells was preceded by the translocation of this factor to the nucleus. The downregulation of TFEB inhibited the induction of autophagy and increased HeLa cell death under AgNP-exposure [73].

**Figure 2:**
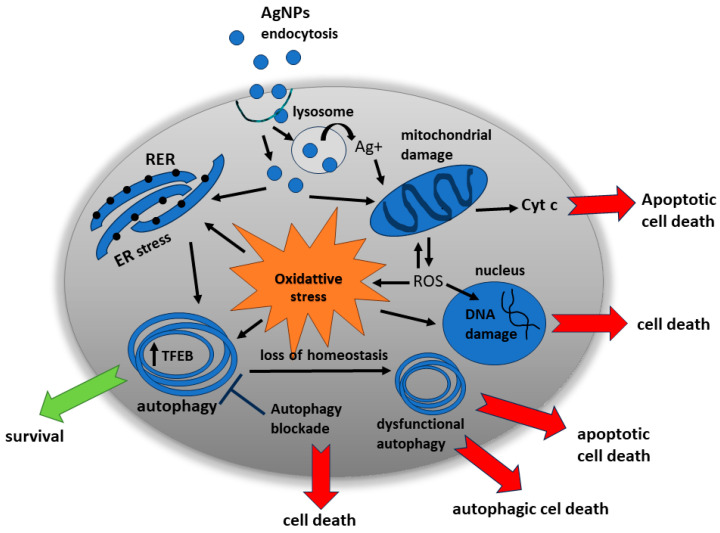
AgNP-induced molecular mechanisms leading to cell survival or death. The dual role of autophagy as a cell protector and cell death executor when the proper functioning of this mechanism is disturbed.

Collected data indicate that autophagy induced under AgNP exposure is not always cytotoxic and the possibility of a dual role of the autophagic mechanism in both normal and cancer cells should be considered (Figure 2).

### 3.5. AgNP-Related Factors of Potential Importance in Determining the Autophagy-Induced Cell Fate

Although a series of studies on AgNP-induced autophagy point to its cytotoxic potential, several in vitro and in vivo studies provide evidence for the protective nature of this mechanism. Therefore, the question is: what are the determinants of the opposite profiles of AgNP-induced autophagy? Since this issue is important in the context of the therapeutic use of AgNPs in cancer treatment, this review attempts to collect and discuss the potential biological factors and NP-related factors that may influence the fate of cells in which autophagic processes have been initiated as a result of exposure to AgNPs. 

The results of the in vitro studies indicate that the concentration of AgNPs is an important factor. In human THP-1 monocytes, AgNPs (15 nm) were found to induce different signatures of ER stress markers in a concentration-dependent manner. Low (10 μg/mL) concentrations of AgNPs induce an ER stress response but not cell death, whereas higher concentrations (25 μg/mL) result in an atypical ER stress response associated with ATF-6 degradation and pyroptotic cell death (an inflammatory form of programmed cell death) through NLRP-3 inflammasome activation [47]. The induction of autophagic flux and enhanced lysosomal activity without apoptotic cell death has been observed in HepG2 cells at noncytotoxic concentrations in the range of 0.1–1 µg/mL AgNPs. Sub-cytotoxic concentrations (<10 µg/mL) were found to enhance the expression of both autophagy-related LC3 and CHOP, an apoptosis inducing ER-stress protein, whereas higher concentrations of AgNPs (10, 25, and 50 µg/mL) induce increased caspase-3 activity associated with apoptosis [6]. The authors suggest a protective role of autophagy at sub-cytotoxic concentrations of AgNPs in HepG2 cells. The results of Zielińska et al. [11] also indicate concentration-dependent viability, proliferation, and death of PANC-1 cells. 

However, other investigations have contradicted the above-mentioned results. AgNPs in a low concentration range of 2–15 μg/mL were found to activate PARP and caspase-3 in mouse embryonic fibroblasts [48] and in HeLa EGFP-LC3 cells; AgNPs of similar size (26.5 ± 8.4 nm) and in a similar concentration range of 2–20 µg/mL were found to induce protective autophagy without disturbing the autophagic flux [50]. In an osteocarcinoma cell line (OS), biosynthesized AgNPs were found to simultaneously induce apoptosis and autophagy in the concentration range of 5–60 µg/mL [51].

The summary of the data prepared for this review (Table 1 and Table 2) suggests that the induction of protective autophagy using in vitro models is usually reported at rather low AgNP concentrations < 10 µg/mL, whereas cytotoxic autophagy is generally demonstrated above this threshold. An interesting point of view has been presented by Li et al. [7] and Fageria et al. [51], who proposed that autophagy is inherently cell protective, and that at higher AgNPs concentrations, autophagy provides a defense mechanism against apoptosis with both processes occurring in parallel.

In addition to the concentration range, the nanoparticle-activated autophagic effects appear to be dependent on the dispersity, size, charge, and surface chemistry of nanoparticles (for a review, see [45]). Higher dispersity and smaller size usually promote the strongest autophagy. Mishra et al. [6] reported that AgNPs can activate autophagic-lysosomal interruption in a size-dependent manner. Small 10 nm AgNPs provide the highest cellular responses when compared with 50 and 100 nm AgNPs, exhibiting the highest uptake in HepG2 cells, which is characterized by extensive accumulation inside the cells, induced autophagy, and enhanced lysosomal activity. Decreased viability, proliferation, and death was also reported to be size-dependent in PANC1 pancreatic ductal adenocarcinoma cells. AgNPs with an average diameter of 2.6 nm were found to induce 16-fold stronger cytotoxicity after 24 h exposure (IC50 = 1.67 μg/mL) relative to 18 nm AgNPs (IC_50_ = 26.81 μg/mL) [11]. Time dependency was also shown to exist in AgNP-treated human breast cancer cells, in which an enhanced autophagic flux occurs immediately after exposure, or inhibited autophagic flux occurs following a significant delay after exposure [12].

In vivo studies using animal models of prolonged exposure to AgNPs have been rare but quite clearly indicate a relationship between AgNP concentration and autophagy-related consequences in tissues of exposed animals. The time course of interrelated changes in markers of energy metabolism (ATP), autophagy (LC3-II), and apoptosis (caspase-3) indicate that, although autophagy is induced early in the liver tissue of rats exposed to a high dose of AgNPs (500 mg/kg b.w.), it fails to preserve energetic homeostasis in hepatocytes and leads to apoptosis with impaired organ function over time [16]. On the other hand, studies of immature rats subjected to a low dose of AgNPs (0.2 mg/kg b.w.) suggest a protective function of ER stress-induced autophagy in the brain tissue of exposed animals [18].

It has been assumed that the induction of autophagy by AgNPs in cancer cells is a dynamic process that is strongly connected with other cellular processes, such as endocytosis. As a result, it can be difficult to simply categorize autophagy as protective or toxic [12]. According to the results of the study, fewer cytotoxic effects were observed in physiological HaCaT cells compared with human breast cancer cells, suggesting that the characteristics of individual cell lines will affect the process of AgNP-induced autophagy, especially in the context of neoplastic processes during which autophagy is abnormal. The importance of the biological condition of the cell, healthy vs. diseased, has been also revealed by Zielińska et al. [11]. Cancerous PANC-1 cells were found to be more sensitive to AgNP-induced cell death than non-cancerous hTERT cells.

Moreover, the biology of the cancer and its genetic profile may also be of significance, as the human OS cell line was more resistant to AgNPs than the hepatocellular carcinoma (HCC) cell line [51]. Similarly, breast cancer cells SKBR3 were more sensitive to the AgNPs-EPS treatment in comparison to HT-29, HCT 116, and Caco-2 colon cancer cell lines [14].

The individual sensitivity of a cancer cell line to the toxic impact of AgNPs can depend on their uptake efficiency by the mechanism of endocytosis, which may be different in various cancers [24]. Data summarized in Table 2 indicate opposing effects of AgNPs in terms of interference with autophagic flux in cancer cell lines. The presence or absence of the blockade of the autophagic pathway appears to be independent of AgNP concentration. Thus, it may depend on the cancer type.

**Figure 3:**
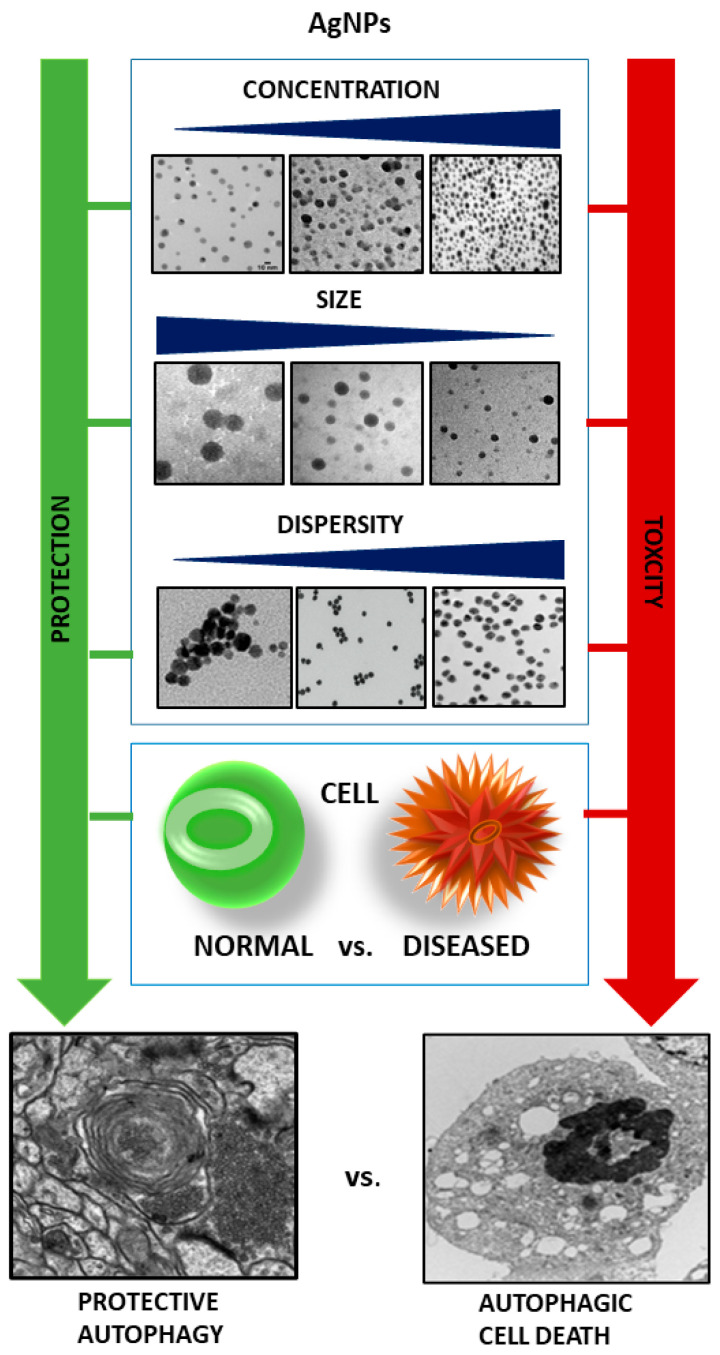
Cellular and AgNP-related factors determining the protective or toxic profile of AgNP-induced autophagy. Low AgNPs concentration, larger size, and agglomeration of nanoparticles favor protective autophagy in normal cells whereas higher AgNPs concentrations, small size, and high dispersity induce autophagy of toxic nature, particularly in diseased cells.

The currently available data summarized in this review indicate that NP-related factors such as size, concentration, and dispersity have significant influences on autophagy-related cellular effects. The cell type (for example, different types of cancer) and the condition of the cell (healthy vs. diseased) may be another important factor influencing AgNP-cell interactions and determining the final outcome of AgNP-induced autophagy (Figure 3).

### 3.6. Potential Medical Implementation of AgNP-Induced Autophagy in Cancer Therapy—Unanswered Questions

Accumulating evidence indicates the involvement of autophagy in the pathomechanisms of various diseases and suggests that the modulation of the regulatory molecules of autophagic machinery may allow us to influence the course of the disease.

Recent studies indicate that metallic nanoparticles are capable of selective overstimulation of autophagy and mitophagy in cancer cells, with the vast majority of nanoparticles inducing death-promoting autophagy [23]. On the other hand, these nanomaterials can modulate autophagy by blocking its physiological flux and act as cytotoxic agents themselves. Nanoparticle-induced dysfunctional autophagy may be associated with lysosomal perturbations that block autophagosome-lysosome fusion, leading to the accumulation of autophagosomes. The link between the exposure to nanomaterials and disruption of lysosomal trafficking and subsequent autophagy may be relevant to AgNP-induced toxicity, which is undesirable in physiological tissues but may be of significance in unhealthy tissues. Therefore, the modulation of autophagy has been proposed as a therapeutic approach in the treatment of various types of cancers [74] and the concept of using AgNPs in this process has been suggested.

Both the unique architecture of the tumor vasculature with a fenestrated endothelial layer and impaired lymphatic drainage facilitate the penetration and accumulation of nano-materials within cancerous tissues. AgNPs can passively penetrate the tumor tissue, accumulating inside in high concentrations via a mechanism known as the enhanced permeability and retention effect (EPR), which is optimized by the shape, size, and functionalization of the surface [75]. Active targeting has also been proposed to increase the cancer specificity of AgNPs by binding to surfaces of cancer-specific antibodies or receptor ligands, or by delivering AgNPs as active components of complex nanosystems (reviewed in [24]). In addition, AgNPs exert anti-angiogenic effects by the inhibition of the vascular endothelial growth factor (VEGF), thus contributing to tumor volume reduction.

The high anti-cancer potential of AgNPs has been previously reported, which is based on various toxic mechanisms (for a review, see [76]. One of the toxic effects of AgNPs on cancer is the overproduction of ROS that can induce damage to crucial biomolecules, followed by apoptotic cell death. The anti-proliferative effect of AgNPs has been shown in H1299 human lung cancer cells [77], SCC-25 human tongue squamous carcinoma cells [78], and MCF-7 breast cancer cells [79]. Biosynthesized protein-capped AgNPs were found to sensitize cancer cells of two different origins, epithelial HCC hepatocellular carcinoma cells and mesenchymal OS human osteosarcoma cells, with acquired resistance to cisplatin by enhancing the cytotoxicity of this chemotherapeutic drug [51].

Cancer cells are characterized by higher sensitivity to oxidative stress compared to healthy cells, and due to their rapid metabolism and intensive proliferation, they require high levels of ATP [80]. These features favor the abnormal autophagy activity in tumor cells. For this reason, tumor cell survival may be affected by the inhibition of the autophagy process, or alternatively, the excessive induction of autophagy by autophagy inducers may also lead to autophagic death of tumor cells. Thus, AgNP-based anticancer therapy would rely on the capability of AgNPs to induce dysfunctional autophagy as a mechanism of cellular toxicity. However, this review shows that the interference of AgNPs with autophagic flux is not a common mechanism across different cancer cell lines and appears to be dependent on unknown factors.

Another important issue is that the mechanisms and extent of the adaptation of cancer cells to autophagy inhibition have not yet been determined [38]. It is known that one of the major characteristics of cancer cells is the ability to avoid apoptotic death, which is a well-known mechanism responsible for their survival and progression [81]. From the tumor perspective, the avoidance of apoptosis is a cellular stress response that is induced for cell survival upon exposure to stress stimuli. Resistance to apoptosis contributes to tumor progression and the resistance to chemo-, radio-, and immunotherapies, all of which are based on the activation of cell death pathways, including apoptosis [82]. This raises the question of whether oncologically transformed cells will escape AgNP-induced autophagic cell death following therapy, as it happens in the case of apoptosis.

Another fundamental question in light of the potential use of AgNPs in cancer therapy, is whether the AgNP-induced autophagy pathway will provide protective or cytotoxic functions in transformed cells where the dysregulation of autophagy is an indicative feature. Furthermore, the well-known toxic potential of AgNPs towards all cells and not just cancerous cells, poses the question of the biocompatibility of AgNPs in clinical applications. This question is more important in light of the evidence described in the current review, which indicates that rather high concentrations of AgNPs are effective in inducing death-promoting autophagy. Therefore, it is presumed that the local application of AgNPs to the tumor should be considered rather than a systemic administration, which, however, would limit the use of AgNPs in supporting anti-cancer therapy.

## 4. Concluding Remarks

The data collected and discussed in this review clearly show that autophagy is a major molecular mechanism induced upon the exposure to AgNPs in both normal and cancerous cells. This collection of findings illustrates the dichotomic nature of AgNP-induced autophagy, which can protect cells from nanotoxicity and execute cell death. Unfortunately, the current evidence is insufficient to characterize the conditions defining the cytotoxic but not cytoprotective effects of AgNP-induced autophagy in cells, particularly those transformed oncologically. It is suspected that several physico-chemical characteristics of AgNPs, as well as the tumor type, may be important factors influencing cell survival or death programs during autophagy, but the evidence remains insufficient.

The significant potential of using AgNPs as inducers of autophagic cell death in oncological therapy cannot overshadow the many unknowns and gaps in our knowledge about the mechanisms of this process. In vivo evidence of the anticancer efficacy of AgNPs is unavailable, and therefore, animal models of cancer exposed to AgNPs are required and warrant profound studies. This is an extremely important issue, because research on autophagy machinery should be extended to interactions of AgNPs with the entire tumor microenvironment and not limited to a single cell layer in mechanistic studies.

In conclusion, a better understanding of the molecular mechanisms of autophagy in pathological settings such as AgNP toxicity and in oncological diseases is now a fundamental requirement that should precede the implementation of AgNP-induced autophagy as a safe strategy supporting the treatment of oncological diseases. In particular, the interplay between autophagy and AgNP-mediated cell death should be better explored in cancerous cells in order to identify and define the determinants of the expected therapeutic outcome. The most important area of research with respect to autophagy as a novel molecular target for therapeutic intervention using AgNPs is the development of animal models of cancer in which the advantage of anti-cancer activity and the disadvantage of AgNP cytotoxicity can be examined in detail.

## Figures and Tables

**Table 1:** Markers of AgNP-induced autophagy and cell death based on in vitro studies using physiological cells and cell lines, and in vivo studies in animal models of exposure.

Type of Cell/Tissue	AgNPs		Autophagy Markers	Autophagy Blockade	Cell Death/Apoptosis Markers	Ref.
	Diameter	Concentration/Dose				
IN VITRO						
Human monocytes (THP-1)	30 nm	5, 10 µg/mL	↑LC3-II Autophagosomes	Yes ↑p62	N/A	[46]
Human monocytes (THP-1)	15 nm	(a) 1–5 µg/mL (b) 25 µg/mL	ER stress (a) Atypical ER stress (b)	N/A	Absent (a) Pyroptosis caspase-1 and IL-1β activation (b)	[47]
Mouse hippocampal neuronal cell line (HT22)	20 nm	25, 50, 100 μg/mL	Autophagosomes ↑LC3 II/I	No ↓p62	↑Caspase-3; ↑Bax ↓Bcl-2	[10]
Human embryonic kidney cell (HEK293T)	25 nm (PVP-coated)	2, 4, 6 μg/mL	↑LC3II; impaired lysosome integrity and protease activity	Yes ↑p62	N/A	[8]
Mouse embryonic fibroblasts (NIH 3T3)	26 ± 7.6 nm	2, 5, 10, 15 μg/mL	Autophagosomes, AVOs, ↑LC3-II (all conc. after 18 h)	Yes ↑p62	Nuclear fragmentation; apoptotic cells ↑PARP and ↑caspase-3 (all conc. after 24 h)	[48]
Rat cardiomyoblasts (H9c2)	4–12 nm	3.5 μg/mL	↑Atg5; ↑Beclin1; ↑LC3BII	N/A	Apoptosis (↑DNA fragmentation)	[13]
Mouse mammary epithelial cell line (HC11)	<100 nm	(a) 6.25, 12.5, (b) 25, 50 μg/mL	Autophagosomes ↑LC3-II/I and ↑Beclin-1 ↓MMP	No ↓p62	Cell death (MTT) (b)	[49]
IN VIVO						
Rat brain, neurons	30 nm	32, 80, or 200 mg/kg	↑LC3-II/LC3-I; ↑pULK; ↑beclin-1 (all conc.)	No ↓p62	Ultrastructural changes; ↑caspase-3; ↑caspase-9; ↑PARP (all conc.)	[7]
Rat liver	10–30 nm	500 mg/kg b.w.	Autophagic structures ↑LC3-II	N/A	Apoptosis ↑caspase-3; ↑TUNEL-positive cells	[16]
Neurons; adult rat brain	10 ± 4	0.2 mg/kg b.w.	Mitochondrial elongation; autophagic structures; ↑LC3-II; ↑beclin-1	Yes, lysosomal proteins cathepsin B and Rab7 do not increase	Morphologically not observed, ↓Bax/Bcl-2	[17]
Neurons; immature rat brain	10 ± 4	0.2 mg/kg b.w.	Mitochondrial elongation; autophagic structures	N/A	Morphologically not observed	[18]

N/A—unavailable information; PVP—polyvinylpyrrolidone, coating agent; AVOs—acidic vesicular organelles; MMP—mitochondrial membrane potential.

**Table 2:** Markers of AgNP-induced autophagy and cell death based on in vitro studies using cancer-derived cell lines and animal models of cancer.

Type of Cancer Cell Line	AgNPs		Autophagy Markers	Autophagy Blockade/Defect	Cell Death/Apoptosis Markers	Ref.
	Diameter (nm)	Concentration				
IN VITRO						
Human liver cancer cells HepG2	10, 50, 100 nm	(a) 1 µg/mL (b) 10, 25, 50 µg/mL	↑Lysosomal activity	N/A	(a) No (b) ↑Caspase-3 activity	[6]
Human pancreatic ductal adenocarcinoma (PANC-1)	2.6 nm 18 nm	0.5–3.5 μg/mL (a) 5 μg/mL (b) 10–50 μg/mL	↑LC3-II Autophagosomes Autophagolysosomes ↑LC3-II	N/A	Morphologically—apoptosis, necrosis/necroptosis ↑Early and late apoptosis (Annexin V/PI) ↑Bax; ↓Bcl-2; ↑p53; ↑Necroptosis-related proteins: RIP-3, MLKL (a) Morphologically—apoptosis, necrosis and necroptosis; (b) ↑Early and late apoptosis (Annexin V/PI); ↑Bax, ↓Bcl-2; ↑p53; ↑necroptosis-related proteins: RIP-3, MLKL	[11]
Human cell lines hepatocellular carcinoma (HCC) and human osteosarcoma (OS)	Biogenic bAgNPs 8.0 ± 2.7 nm	15–40 µg/mL	↑Autophagic vesicles ↑Autophagolysosomes ↑LC3B-II expression	N/A	Apoptosis Morphological markers -fragmented nuclei ↑Caspase-3 activity, the cleavage of PARP-1, ↑fragmented DNA	[51]
HeLa cells B16 melanoma cells	AgNPs-PVP 26.5 ± 8.4	10 µg/mL 50 μg/mL	↑Autophagosomes ↑LC3-BII ↑Autophagosomes	No No	Apoptosis/necrosis ↑Caspase-3 enhanced by wortmannin Apoptosis/necrosis enhanced by wortmannin	[50]
A549 human lung adenocarcinoma	60-nm AgNPs		Lysosomal pH alkalization and autophagosome formation. ↑LC3-BII	Yes ↑p62	Cellular damage	[52]
Human breast cancer cells MCF-7 and MDA-MB-468	9 ± 2.2 19 ± 2	(a) 25 mM (b) 100 mM	↑LC3B-II; ↑beclin1; ↑Atg3 (a, b)	No early (6 h) post-exposure Yes after prolonged exposure (24 h) ↑p62; ↑Rab7 ↓LAMP1; ↓AO fluorescence	↓Mitochondrial potential, Apoptosis—AnnexinV/PI ↓PARP expression	[12]
SH-SY5Y	30 nm	12.5 μg/mL	Autophagosomes, acidic autophagic vacuoles, ↑beclin-1; ↑LC3-II	No ↓p62	No	[7]
Human prostate cancer cell line (PC-3)	AgNPs-PVP 78.24 ± 0.58	2, 4, 6 µg/mL	↑LC3-II/LC3-I	Yes ↑p62; Lysosomal degradation ↓cathepsin D ↓lysosome-related genes: CSTA, CSTD, CLCN7, MCOLN1	No apoptotic cells (Annexin-V FITC/PI)	[9]
Breast cancer cells (SKBR3)	N/A	AgNPs-EPS 5 μg/mL	↑ATG5; ↑ATG7; ↑LC3-II; ↑beclin-1	No ↓AKT; ↓p-AKT; ↓p62; ↓HSP90	No No DNA fragmentation	[14]
Lung epithelial cancer cells (A549)	10 to 20 nm,	32.33 μg/mL	Autophagosomes and autolysosomes ↑ATG5; ↑LC3-II	Yes ↑p62,	Activated caspase-3	[15]
Colorectal adenocarcinoma cell line (HT-29)	<100 nm	33.45 μg/mL	↑Beclin-1; ↑XBP-1; ↑CHOP; ↑LC3-II	N/A	Apoptosis; ↑cyt-C; ↑p53; ↑ Bax; ↑ASP3, ↑CASP8; ↑CASP9; ↑CASP12	[53]
IN VIVO						
B16 cells injected to the C57BL/6 mice	AgNPs-PVP	1.5 mg/kg Ag NPs, 1.5 mg/kg Ag NPs plus 25 nmol/kg wortmannin.	↑LC3-II; Inhibited by wortmannin	No	↓Tumor growth ↑Apoptosis (TUNEL) Enhanced by wortmannin	[50]

AO—acridine orange the marker of lysosome stability; PI—propidium iodide; AgNPs-EPS—AgNPs coated with exopolysaccharides; AgNPs-PVP—AgNPs coated with polyvinylpyrrolidone; N/A—not available.

## Data Availability

Not applicable.
